# Proteogenomic analysis integrated with electronic health records data reveals disease-associated variants in Black Americans

**DOI:** 10.1172/JCI181802

**Published:** 2024-09-24

**Authors:** Usman A. Tahir, Jacob L. Barber, Daniel E. Cruz, Meltem Ece Kars, Shuliang Deng, Bjoernar Tuftin, Madeline G. Gillman, Mark D. Benson, Jeremy M. Robbins, Zsu-Zsu Chen, Prashant Rao, Daniel H. Katz, Laurie Farrell, Tamar Sofer, Michael E. Hall, Lynette Ekunwe, Russell P. Tracy, Peter Durda, Kent D. Taylor, Yongmei Liu, W. Craig Johnson, Xiuqing Guo, Yii-Der Ida Chen, Ani W. Manichaikul, Deepti Jain, NHLBI Trans-Omics for Precision Medicine Consortium, Thomas J. Wang, Alex P. Reiner, Pradeep Natarajan, Yuval Itan, Stephen S. Rich, Jerome I. Rotter, James G. Wilson, Laura M. Raffield, Robert E. Gerszten

**Affiliations:** 1 Division of Cardiovascular Medicine, Beth Israel Deaconess Medical Center, Harvard Medical School, Boston, Massachusetts, USA.; 2 Mount Sinai School of Medicine, New York, New York, USA.; 3 University of North Carolina School of Medicine, Raleigh, North Carolina, USA.; 4 Stanford School of Medicine, Palo Alto, California, USA.; 5 University of Mississippi Medical Center, Jackson, Mississippi, USA.; 6 Department of Pathology Laboratory Medicine, Larner College of Medicine, University of Vermont, Burlington, Vermont, USA.; 7 The Institute for Translational Genomics and Population Sciences, Department of Pediatrics, The Lundquist Institute for Biomedical Innovation, Torrance, California, USA.; 8 Department of Medicine, Division of Cardiology, Duke Molecular Physiology Institute, Duke University Medical Center, Durham, North Carolina, USA.; 9 Department of Biostatistics, University of Washington, Seattle, Washington, USA.; 10 Center for Public Health Genomics and; 11 Division of Biostatistics and Epidemiology, Department of Public Health Sciences, University of Virginia, Charlottesville, Virginia, USA.; 12 University of Washington, Seattle, Washington.; 13 The NHLBI Trans-Omics for Precision Medicine Consortium is detailed in Supplemental Acknowledgments.; 14 Department of Medicine, UT Southwestern Medical Center, Dallas, Texas, USA.; 15 Cardiovascular Research Center, Massachusetts General Hospital, Harvard Medical School, Boston, Massachusetts, USA.; 16 Broad Institute of Harvard and MIT, Cambridge, Massachusetts, USA.

**Keywords:** Genetics, Immunology, Genetic diseases, Population genetics, Proteomics

## Abstract

**BACKGROUND:**

Most GWAS of plasma proteomics have focused on White individuals of European ancestry, limiting biological insight from other ancestry-enriched protein quantitative loci (pQTL).

**METHODS:**

We conducted a discovery GWAS of approximately 3,000 plasma proteins measured by the antibody-based Olink platform in 1,054 Black adults from the Jackson Heart Study (JHS) and validated our findings in the Multi-Ethnic Study of Atherosclerosis (MESA). The genetic architecture of identified pQTLs was further explored through fine mapping and admixture association analysis. Finally, using our pQTL findings, we performed a phenome-wide association study (PheWAS) across 2 large multiethnic electronic health record (EHR) systems in All of Us and BioMe.

**RESULTS:**

We identified 1,002 pQTLs for 925 protein assays. Fine mapping and admixture analyses suggested allelic heterogeneity of the plasma proteome across diverse populations. We identified associations for variants enriched in African ancestry, many in diseases that lack precise biomarkers, including *cis*-pQTLs for cathepsin L (CTSL) and Siglec-9, which were linked with sarcoidosis and non-Hodgkin’s lymphoma, respectively. We found concordant associations across clinical diagnoses and laboratory measurements, elucidating disease pathways, including a cis*-*pQTL associated with circulating CD58, WBC count, and multiple sclerosis.

**CONCLUSIONS:**

Our findings emphasize the value of leveraging diverse populations to enhance biological insights from proteomics GWAS, and we have made this resource readily available as an interactive web portal.

**FUNDING:**

NIH K08 HL161445-01A1; 5T32HL160522-03; HHSN268201600034I; HL133870.

## Abstract 2

Our study integrates genetic and protein associations with large electronic health record datasets in diverse populations to enhance biological insights in clinical disease.

## Introduction

Large-scale GWAS have uncovered thousands of loci implicated in human disease. However, genotype is often far removed from phenotype, limiting insight into the pathological processes that result in disease. Proteins are the main effectors of many biological processes and are a closer proxy to observed phenotypes. The integration of genetics with plasma proteomics has been helpful in bridging the gap between genotype and phenotype by uncovering genetic determinants of circulating proteins, illuminating biological effectors of complex disease, and even suggesting potential therapeutic targets.

Expanded proteomic platforms have enabled the profiling of thousands of circulating factors for integration with genome-wide genetic data (1–11). Recently, Sun et al. performed the largest ever proteomics GWAS in over 50,000 individuals using the 3,000 protein OLINK platform (https://olink.com/), identifying many protein quantitative loci (pQTLs) with shared genomic signals across a wide range of phenotypes (3). To date, however, most such analyses, including that of Sun et al., have primarily focused on White individuals of European ancestry. This limits the generalizability of findings across other populations, where the genetic architecture of pQTLs may differ due to varying linkage disequilibrium (LD) patterns, allele frequencies, and effect sizes of causal variants. Moreover, the limited diversity in these studies restricts our ability to gain insights from variants that are ancestry-enriched. As a proof of concept, we previously performed a GWAS of plasma proteomics using the aptamer-based SOMAlogic platform in a Black population from the Jackson Heart Study (JHS). We identified proteomic associations for ancestry-enriched SNPs in genetic loci associated with clinical disease including *TTR* (amyloidosis), *APOL1* (kidney disease), and *HBB* (sickle cell disease) among other findings (1, 12).

Considering large-scale ongoing efforts in predominantly White populations (3, 8), we posited that additional proteogenomic studies focused in more diverse populations would enhance the yield of biological insights. To this end, we conducted a genetic discovery using whole-genome sequencing for determinants of plasma proteins measured by the antibody-based Olink 3K platform in self-reported Black adults from JHS. Based on genetic similarity to the 1000G reference panel (13), these participants have on average 82% African ancestry, suggesting we are powered to discover variants that are rare in European and common in African reference populations. We attempted replication of our findings in the Multi-Ethnic Study of Atherosclerosis (MESA), including both *cis* and *trans* genetic signals, many of which are more common in African versus European reference populations. We performed statistical fine mapping and local admixture analyses of our genetic signals to assess for allelic heterogeneity in plasma proteome across European and African ancestries. Given the Eurocentric bias of most published GWAS and commonly used phenome-wide summary statistics from UK Biobank to date (14), we hypothesized that pQTLs derived from the Black population in JHS would identify new disease associations. We examined the clinical relevance of our pQTLs in a phenome-wide association study (PheWAS) using a healthcare-derived resource consisting of disease-enriched populations (BioMe) (15) and a multi-ethnic biobank (All of Us) (16). Our study is the first, to our knowledge, to integrate large-scale pQTL analyses with these diverse electronic health record (EHR) datasets. We tested for pQTL associations across a range of clinical phenotypes and extensive laboratory studies, many never previously assessed, highlighting associations in African ancestry–enriched variants. Our proteogenomic study thus examined the value of leveraging diverse populations to gain insight into clinical disease biology.

## Results

**Figure 1:**
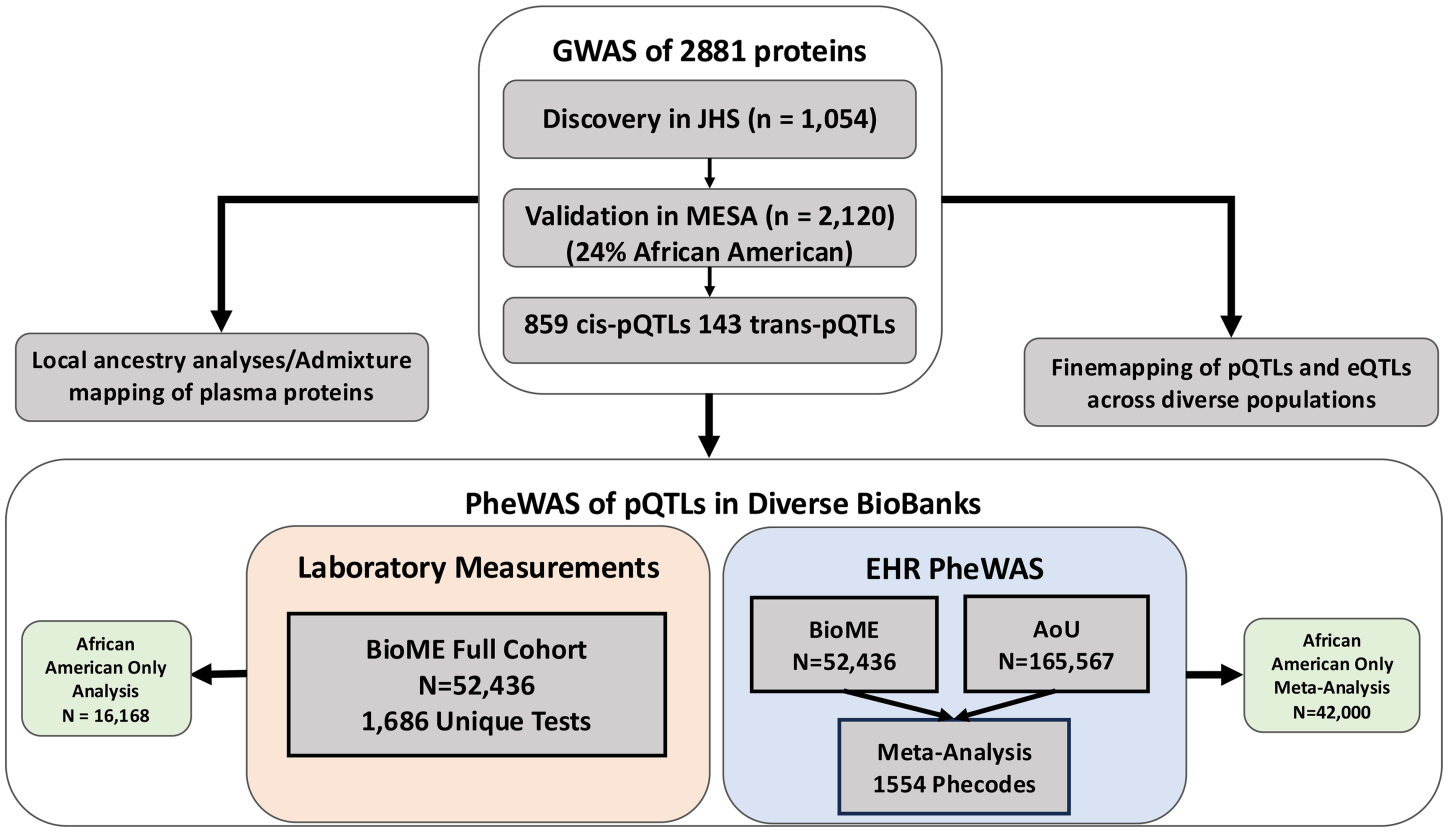
Study design. We performed discovery GWAS of 2,881 plasma proteins in the JHS (*n* = 1,040) and validated associations in the MESA (*n* = 2,120). pQTLs were interrogated in 2 biobanks of diverse individuals for phenotype associations through PheWAS. AoU, All of Us research program.

**Figure 2:**
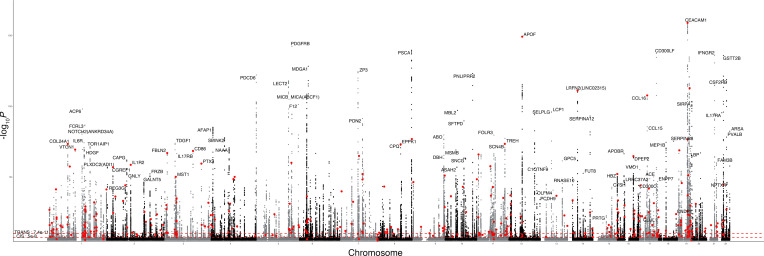
Genetic associations with plasma abundance of 2,881 proteins in Black individuals from the JHS (Manhattan plot). Significance thresholds reflect genome-wide significance for cis-pQTLs (*P* < 5 × 10^–8^) or Bonferroni’s significance for trans-pQTLs (*P* < 7.7 × 10^-11^). pQTLs enriched in African ancestry (NFEs MAF < 1% based on gnomAD) are colored in red. Trans-pQTLs are labeled with the protein name and gene name.

Leveraging the overall study design summarized in Figure 1, we first performed GWAS for 2,881 proteins in 1,054 self-identified Black individuals from JHS (63% women, 37% men; Supplemental Table 1; supplemental material available online with this article; https://doi.org/10.1172/JCI181802DS1) using approximately 28 million variants with a minor allele count greater than 5. Heritability was estimated using related JHS individuals from 250 families, adjusting for age and sex in SOLAR (17). Mean estimated total heritability across all proteins was 26% (Supplemental Table 2). We identified 859 *cis*-pQTLs (*P* < 5 × 10^–8^) and 143 *tran*s-pQTLs (7.7 × 10^–11^) representing 925 unique Olink assays, 939 sentinel SNPs, and 892 corresponding genes (Figure 2). Of the 1,002 pQTLs identified in JHS, 86% replicated in the multiethnic MESA cohort (*n* = 2,120) with a Bonferroni’s corrected *P* value (1002 tests) of less than 5 × 10^–5^ and 96% replicated at nominal *P* < 0.05 with the same direction of effect; 317 (34%) of the unique sentinel SNPs were rare in non-Finnish Europeans (NFEs) (reference population gnoMAD, version 3; ref. 18) and 387 (41%) had a MAF in NFE less than 5%. While there were many overlapping association regions identified in the UK Biobank proteomics GWAS, our extensive catalogue of variants enriched in individuals of African ancestry allowed us to test for their phenotypic associations in Black populations, as described below.

**Figure 3:**
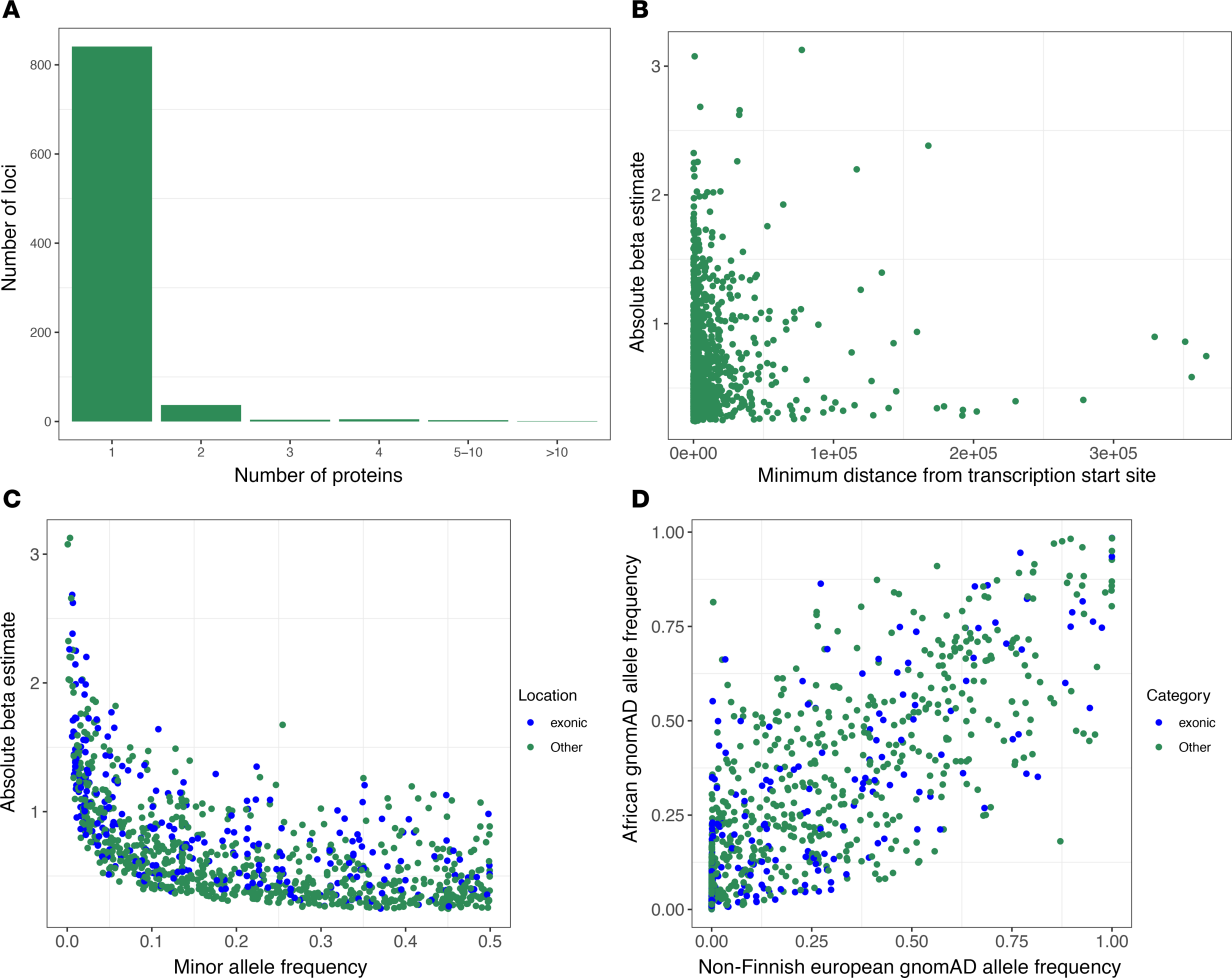
Genetic architecture of plasma pQTLs. (**A**) The number of proteins significantly associated with each sentinel pQTL. (**B**) Distance of the sentinel variant from the transcription start site versus the effect size of the variant on protein abundance for cis pQTL loci. (**C**)Minor allele frequency of pQTLs in JHS versus estimated effect size. Points are colored based on genomic location (exonic or intronic). (**D**)pQTL MAF in NFEs and MAF in individuals of African ancestry. Points are colored based on genomic location (exonic or intronic) as denoted in the label.

**Figure 4:**
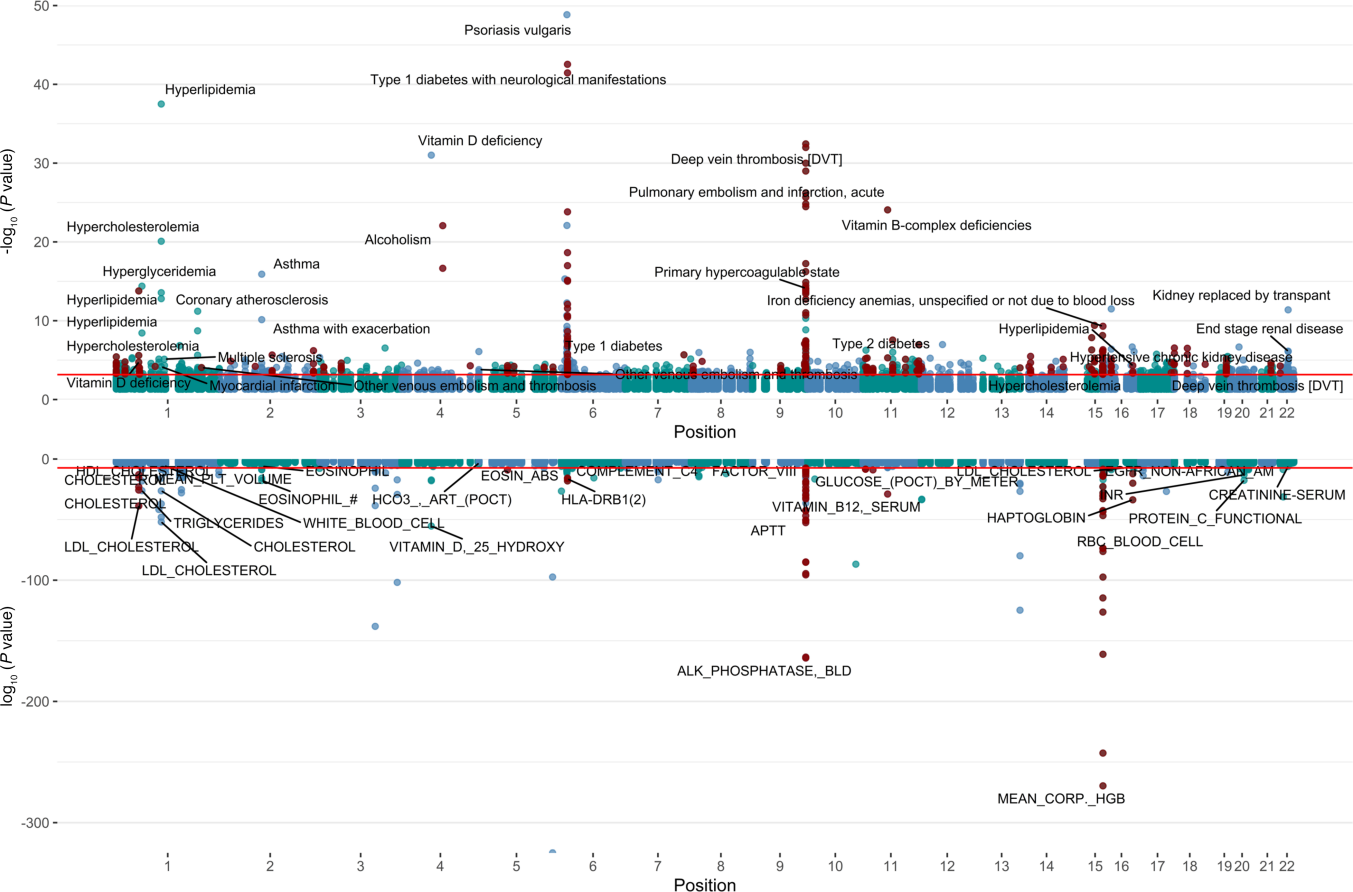
Miami plot representing pQTL associations with binary phecodes and continuous laboratory measurements in BioMe and All of Us. Top: pQTL associations with binary phecodes. Bottom: continuous laboratory measurements. Each point represents a unique phenotypic association for a given pQTL; points colored in red are those with MAF in NFE of less than 1%. Red lines represent FDR significance.

Consistent with prior studies (1–3), the most pleiotropic gene region was *ABO* associated with 29 proteins, followed by *F12* and *FUT2*, which were associated with levels of 7 proteins each (Figure 3). Proteins with the most pQTLs were SERPINI2 (4), CTRC (3), MUC2 (3), and PON3 (3). Of the sentinel SNPs of our pQTLs, 25% were exonic and 46% intronic. Utilizing the sorting intolerant from tolerant (SIFT) algorithm (19), 74 missense variants were predicted to be deleterious. These included variants in well-known disease loci such as *TTR* V1221, associated with cardiomyopathy and neuropathy in Black individuals. We also found variants predicted to be deleterious that are rare in White individuals (gnomAD NFE MAF <1%) and understudied in their association with clinical disease due to the Eurocentric focus of most GWAS to date, including *cis*-pQTLs in *MMP10* and *COLEC12*. These variants revealed new links to clinical phenotypes in diverse biobanks, as described below. (Figure 4 and Supplemental Tables 9–12).

In addition to the *cis*-pQTLs that map to the cognate gene for the circulating protein, we identified 143 *trans*-pQTLs, of which 29 had sentinel variants that were African ancestry enriched. These include biologically plausible associations of the Duffy variant with the chemokines CCL14, CCL7, and CLEC4A and of the haptoglobin locus with GALNT2, HBQ1, and SERPIND1. Additionally, we found *trans*-pQTLs for proteins and their receptors/binding partners including IL-18 and IL18Bp and PLAU and PLAUR (Table 1).

### Fine mapping and admixture analyses identify allelic heterogeneity for circulating proteins in a multiethnic cohort.

Multiancestry fine mapping can improve the resolution for identifying causal variants in pQTL analyses (20). We first conducted a fixed effects metaanalysis across JHS and MESA (Supplemental Table 4). We then performed statistical fine mapping, yielding 894 protein assays with significant credible sets (Supplemental Table 5). We found that 43% of our protein assays had pQTL credible sets that were distinct from that of the UK Biobank discovery cohort (Supplemental Table 6), highlighting allelic heterogeneity of the plasma-proteomic associations between the study populations. We also performed admixture mapping analysis, leveraging differences in allele frequencies among the ancestries of admixed populations, to identify associations between local African ancestry and protein levels that may be independent of prior GWAS findings. Of the 2,881 proteins assayed on the Olink proteomics platform, 55 proteins showed statistically significant signals of association with regions of local African ancestry (*P* < 3.1 × 10^–08^) in admixture analyses. We attempted replication in 471 self-identified Black participants in MESA with proteomic and genetic data. All 56 protein associations replicated with *P* < 0.05 and consistent direction of effect (Supplemental Table 7). Our admixture mapping identified many previously unreported ancestry-associated regions, including genomic associations with angiotensin-converting enzyme (ACE), neurofascin (NF), and CD33, among others. To assess whether identified association regions were statistically distinct from genome-wide significant associations, we conditioned our findings on the sentinel SNP from our JHS analyses and the UK Biobank discovery cohort. Approximately 20% of the admixture signals remained associated with protein levels, suggesting that there are variants affecting the levels of circulating proteins that were independent of variants from single-variant GWAS.

### Overlap between pQTLs and expression quantitative loci in peripheral blood mononuclear cells in JHS.

To begin to assess functional consequences of our findings, we sought to determine whether our pQTLs were also expression quantitative loci (eQTLs). Given the scarcity of transcriptomics data for individuals of African ancestry in publicly available resources such as GTEx (https://gtexportal.org/home/), we leveraged RNA-Seq of peripheral blood mononuclear cells (PMBCs), performed in a subset of genotyped individuals in JHS. After fine mapping, we found 141 pQTLs with credible sets that overlapped those of either eQTLs or splicing QTLs from JHS PBMCs (sQTLs; Supplemental Table 8). Our work provides a valuable resource to probe the potential regulatory effects of pQTLs, which is in particular vital given the scarcity of transcriptomic datasets in Black individuals.

### PheWAS identified pQTL associations across extensive phenotype and laboratory studies.

We next performed phenome-wide association studies (PheWAS) in over 210,000 individuals from 2 diverse biobanks: BioME and the All of Us research program, across 1,554 distinct EHR-based phecodes. Full cohort metaanalysis identified 511 significant (FDR < 5% with concordant direction of effects, and *P* < 0.05 in both cohorts) pQTL-phecode associations (Supplemental Table 9). Of these associations, 46% included variants rare in individuals of NFE ancestry from gnomAD. Comparison of significant PheWAS findings with the GWAS catalog (21) suggested that 350 of the 511 associations had not been reported in the GWAS catalogue, 177 of which were with variants rare in individuals of NFE. These included associations for *cis*-pQTLs for inter-α trypsin inhibitor 1 (ITIH1) and type I diabetes and Siglec-9 linked with non-Hodgkin’s lymphoma. SNP-phenotype associations were also identified beyond phecodes and blood-based biomarkers using EHR data. For example, we found pQTL associations across a range of clinical diagnostic tests. This includes an association of a *cis*-pQTL for CD36 with QT interval measurements on the electrocardiogram, consistent with a recent multiancestry GWAS of QT intervals (22). Additionally, we found *cis*-pQTLs for fetuin B (FETUB) and tripeptidyl peptidase (TPP1) associated with measurements of forced expiratory volume in pulmonary-function studies. Secondary metaanalysis in self-identified Black individuals yielded largely similar results with concordant directions of effects (Supplemental Table 11; 112 pQTL-phecode associations using FDR <5%) including ancestry-enriched *cis*-pQTLs for annexin II (ANXA2) associated with *Heliobacter pylori* infection and matrix metalloproteinase 10 (MMP10) associated with cardiac conduction disorders. We also found associations for relatively rare diseases that lack precise biomarkers, including a *cis*-pQTL for cathepsin L (CTSL) associated with sarcoidosis, highlighting the value of proteogenomic studies and PheWAS in uncovering potential novel biology for less common phenotypes.

Integration of 1,686 hospital-based laboratory tests from BioMe provided additional context for pQTL-disease associations, through intermediate clinical risk markers (Figure 4 and Table 2). We found 698 FDR significant associations between pQTLs and clinical laboratory tests (Supplemental Table 10). Several of our *cis*-pQTLs align with hospital-based laboratory measurements of the same protein, underscoring the specificity of our proteomics assay and strengthening the pQTL–clinical disease associations. For instance, a *cis*-pQTL for α amylase 2A (AMY2A) was associated with blood amylase in BioMe and primary biliary cirrhosis in our PheWAS. Additionally, pQTLs associated with a pathway of disease (i.e., related protein, laboratory measurement, and disease outcome) may represent particularly attractive targets for functional follow-up. For example, a *cis*-pQTL for interleukin 1 receptor like 1 (IL1RL1), a cytokine that helps mediate allergic immune responses (22), was associated with eosinophil counts and asthma (Table 2). We found an ancestry-enriched *cis*-pQTL for Siglec-9, an immune checkpoint molecule, associated with non-Hodgkin’s lymphoma and urine paraprotein percentage. A *cis*-pQTL for CD58 was associated with multiple sclerosis (MS) as well as WBC count. We conducted a Mendelian randomization analysis leveraging the *cis*-instrumental variable for CD58 in the International Multiple Sclerosis Genetics Consortium (23) and found additional evidence for a potential causal role of circulating CD58 in MS development (Table 3).

## Discussion

Here we present a GWAS of approximately 3K proteins measured using the Olink antibody-based proteomic platform in a self-identified Black population from JHS and validate our findings in MESA. We previously performed GWAS using the SOMALogic 1.3K assay and identified many ancestry-specific variants. In our current study, we expanded our protein coverage using the Olink 3K platform and integrated genetics with an additional approximately 2,200 unique protein assays. We highlight allelic heterogeneity of the plasma proteome across populations of European and African ancestries. Capitalizing on our ancestry-enriched pQTLs, we identified many potentially novel clinical associations in a PheWAS in large and diverse biobanks, enriched for clinical conditions and events. We leveraged an extensive database of laboratory studies from the BioMe healthcare cohort, many of which were never previously analyzed in PheWAS. We found concordance between laboratory measurements and clinical phenotypes, bolstering the validity of our findings and potentially highlighting new biology. In a relatively modest discovery population of Black individuals, our GWAS findings underscore the importance of genetic diversity, especially for variants nearly absent in the European population, to uncover clinically relevant findings.

The integration of pQTL data with clinical phenotypes can help elucidate biological pathways to disease and potential novel risk markers. This is especially valuable for ancestry-enriched variants and less common diseases where datasets are far more limited. While most efforts to integrate pQTL and clinical data derive from cohorts of predominantly White individuals (e.g., UK Biobank), our study is the first, to our knowledge, to integrate large-scale pQTL analyses with All of Us and BioMe, EHR datasets with considerable population diversity. Additionally, we conducted secondary analyses in self-identified Black participants only. There was general concordance between PheWAS findings in the full cohort and in the Black-only analysis, suggesting PheWAS findings from the full cohort are likely not confounded by variations in disease diagnosis by self-reported race and due to social and environmental exposures, including potential discrimination in the healthcare setting. Here, we highlight several clinical findings with ancestry-enriched variants.

We found a *cis*-pQTL in *CTSL*, rare in individuals of NFE ancestry, to be associated with sarcoidosis in the Black-only analysis. Sarcoidosis is an inflammatory condition with systemic effects that may result in severe complications (e.g., cardiac sarcoidosis with heart failure and arrhythmias) that is more prevalent and severe in Black individuals (24). Importantly, reliable biomarkers for diagnosis and prognosis in this disease are lacking. CTSL is a lysosomal protease that is actively secreted in inflammatory processes, and based on limited mouse models of sarcoidosis, it may affect granuloma formation (25). Further studies are needed to assess the role of CTSL both in the pathogenesis and as a biomarker of sarcoidosis in diverse human cohorts. As an additional example highlighting diseases more common in Black individuals, the *TTR* V122I variant — a variant present in 3%–4% of Black individuals — was associated with cardiomyopathy, sinus node dysfunction, and peripheral neuropathy in our PheWAS. This was consistent with the established clinical findings of hereditary *TTR* cardiac amyloidosis. Surprisingly, we found an association of the V122I TTR pQTL with primary angle closure glaucoma. There have been reports of glaucoma associated with amyloid previously, presumably due to amyloid fibrils, resulting in increased intraocular pressure (26). However, glaucoma has not been a prominent feature in prior descriptions in individuals with the *TTR* V1221 variants, who are particularly susceptible to developing cardiovascular and neurologic disease.

An ancestry-enriched *cis*-pQTL for ITIH1, a serum protease primarily expressed in the liver, was associated with type I diabetes. IITIH1 is a binding partner of hyaluronan, a key component of extracellular matrix that is found to be elevated in pancreatic islets of type I diabetics (27) and implicated in the pathogenesis of several autoimmune conditions (28). Further, increased liver expression of ITIH1 has been correlated with markers of insulin resistance and diabetes in humans (29), and serum levels were associated with diabetic retinopathy in prior studies in a small cohort (30). Intriguingly, a nearby pQTL for another member of the ITIH family, ITIH4, was associated with retinal vascular changes in our PheWAS. While the exact functions of ITIH proteins have not been fully elucidated, our findings support a potential role for this family of proteins in the pathogenesis of insulin resistance, type I diabetes, and its complications. We also identified potentially novel PheWAS associations for diseases more common in the general population. An ancestry-enriched *cis*-PQTL in ANXA2 was associated with *Helicobacter pylori* infection. Annexins are phospholipid-binding proteins expressed on epithelial cells and found to be overexpressed in gastric cancer associated with *H*. *pylori* infection in humans (31). Further, it has been suggested that binding of *H*. *pylori* to cellular annexins may help it to evade host immune responses (32). Our PheWAS findings support a mechanistic link between ANXA2 and *H*. *pylori* in humans.

The availability of laboratory measurements, as intermediate phenotypes in BioMe, complementing the EHR-based PheWAS, allowed us to examine the associations among pQTLs, clinical testing, and disease outcomes. This unique resource provided an opportunity to elucidate potential pathways of disease risk involving genetic variation as well as to clarify biological mechanisms involving known disease loci with unknown functions. As an example, the *FAM234A* locus includes African ancestry–enriched variants, previously shown to be associated with RBC traits (33). We found a *trans*-pQTL in this locus for AHSP α hemoglobin stabilizing protein (AHSP) that is associated with RBC width and mean corpuscular volume as well as phecodes related to anemia and hemoglobinopathies. AHSP is involved in the regulation of hemoglobin synthesis and is central to erythropoiesis. This locus’ association with RBC traits may be a reflection of ineffective erythropoiesis and subsequent anemia, mediated by AHSP. In another example, we found a *cis*-pQTL in α amylase (*AMY2A*), associated with laboratory blood amylase levels and primary biliary cirrhosis. While amylase is commonly elevated in conditions causing biliary and pancreatic obstruction, the genetic association with amylase levels and primary biliary cirrhosis suggests a possible causal role of this protein in biliary pathology.

MS is a degenerative disease of the central nervous system leading to debilitating neurological defects. While well known to be associated with immune dysregulation (34), the specific pathways are not well defined. In prior studies, variants in the *CD58* gene were associated with MS, and the presence of *CD58* polymorphisms was found to correlate with increased disease activity in MS (35). We found a *cis*-pQTL in *CD58* that is associated with WBC count as well as MS in both PheWAS and Mendelian randomization (MR) studies. CD58 is widely expressed on WBC cells and in particular contributes to enhanced T cell activity. Dysregulated T cell activity and function are hallmarks in the pathogenesis of MS (36), and our pQTL associations of CD58 with MS in large EHRs suggest a causal link to MS and potentially a biomarker for disease severity and therapeutic responses.

We found a *cis*-pQTL for Siglec-9 that is rare in NFE populations associated with non-Hodgkin’s lymphoma. Siglecs are a family of proteins expressed on myeloid and T cells and function to promote cell-cell interactions while playing a prominent role in inflammatory and immune pathways (37). Further, siglecs are immune checkpoint proteins that bind sialic acids on glycoproteins on tumor cell membranes and that can modulate the immune response by promoting tumor immunity. This pQTL locus has previously been associated with circulating CD5 (38), a glycoprotein expressed on T cells. Aberrant CD5 expression is a hallmark of several subtypes of non-Hodgkin’s lymphomas including mantle cell lymphoma (39). In addition to the association with non-Hodgkin’s lymphoma, we found this pQTL to be associated with urine paraprotein percentage in BioMe. Paraproteinemia, or monoclonal gammopathy, is present in several types of non-Hodgkin’s lymphoma including lymphoblastic lymphoma (40). Our PheWAS associations across both clinical disease and laboratory measurements provide genetic support for Siglec-9 as a potential therapeutic target in non-Hodgkin’s lymphoma.

Genetic analyses in recently admixed populations, characterized by the contribution of 2 or more ancestral groups to the genetic architecture of a population, present an opportunity to identify genetic association regions driven by variants, potentially rare, with different frequencies across ancestral populations. Admixture mapping can increase power to identify novel variants to elucidate the biological mechanisms that enhance disease susceptibility in a population. As an example, African Americans, an admixed population of predominant African and European ancestry, have a higher prevalence of end-stage renal disease (ESRD). Admixture mapping for ESRD in this population discovered an association between the levels of local African ancestry on a region of chromosome 22 and ESRD (41). This genetic signal was later mapped to *apolipoprotein L1* (APOL1), where genetic variants are under positive selective pressure for protection against *Trypanosoma brucei rhodesiense*, a parasite that causes African sleeping sickness (42). Though admixture mapping has been a valuable genomics tool, it has only recently been studied in the context of large-scale omics profiling (43), where intermediate and quantitative phenotypes may improve power for discovery. In our study of the Olink 3k platform, we identify several associations with local African ancestry that were independent of lead SNPs from GWAS in JHS and UK Biobank, including an association in the *cis*-region for CD33. CD33 is a myeloid differentiation antigen expressed on acute myeloid leukemia cells and a therapeutic drug target. Local ancestry signals are driven by variants with highly differentiated allele frequencies across reference populations. When such signals are independent of standard GWAS variants, this may indicate a more complex or polygenic genetic architecture in the region, an important consideration when assessing the phenotypic effects of genetically mediated levels of proteins using genetic instruments in MR studies (44). Our admixture analyses, along with our fine mapping results, reinforce the concept of allelic heterogeneity within the plasma proteome among different populations.

### Limitations.

In our discovery cohort in JHS, we identified pQTLs enriched in African ancestry despite the relatively modest sample size. Larger samples sizes may enhance discovery efforts in addition to downstream analyses such as statistical fine mapping to identify credible sets of potential causal variants. When feasible, downstream in silico methods including colocalization and MR will also be helpful to assess potential causal relationships between proteins and disease. Our study measured protein levels at a single time point. Serial protein measurements over time could improve measurement accuracy and enhance the power for detecting associations. Future GWAS studies are needed to assess whether the genetic determinants of protein level changes over time differ from those observed at baseline. While our previous work has demonstrated good correlations between Olink and select ELISAs (12), we note that future work to validate QTLs from discovery platforms such as Olink using orthogonal methods (i.e., ELISA, mass spectrometry) will be valuable.

Rigorous protocols were used for sample collection and storage, though we performed proteomic analyses on samples that had been archived for varying time periods. However, both JHS and MESA conducted their baseline examination within a similar 2- to 4-year period. This consistency in sampling periods reduces the likelihood of temporal biases affecting the protein measurements. We also excluded proteins with high coefficient of variation (CV) (>20%; only 60 out of approximately 3k proteins) from our analyses to ensure the reliability of our results. Finally, our work strongly motivates mechanistic studies in model organisms to further elucidate underlying pathways, particularly in instances where clinical PheWAS outcomes were strongly corroborated by laboratory findings (e.g., an inflammatory disease with altered circulating WBC count or a thrombotic disease associated with altered coagulation parameters).

In our PheWAS, we conducted a sensitivity analysis to assess whether findings from our metaanalyses were consistent within the African American cohort or potentially influenced by confounding factors such as social and environmental exposures, including potential discrimination in the healthcare setting. We acknowledge that race is a social construct and the use of more detailed social determinants of health measures, for example, metrics of discrimination and racism and of area and individual level socioeconomic factors, among others, would strengthen efforts to account for these confounding factors (45). However, the available EHR and biobanks are limited in their ability to capture the full spectrum of social determinants of health variables that may be relevant here. Future studies are needed to better capture these important factors. The objective of our genetic analyses was to leverage the genetic diversity within our cohorts (18) to include variants that are rare or absent in individuals of NFE ancestry (noting that individuals with similarity to European reference panels are dramatically overrepresented in pQTL studies so far) for biological insights into clinical disease. We do not attempt to identify differential associations across race and ethnicity as social constructs; we do, however, report differences in genetic allele frequencies by population in external reference populations (notably gnomAD) for many of our identified lead signals, highlighting the importance of pQTL studies across many global populations to capture pQTL signals with population-differentiated frequencies.

The PheWAS approach relies on the use of phecodes within an electronic health system, which may introduce spurious associations (in addition to false negatives) due to diagnostic errors and bias. However, we incorporate laboratory values in addition to our PheWAS and show concordance for genetic associations across diagnoses codes and lab values, in 2 separate 2 EHR databases, which helps to mitigate healthcare system biases.

### Conclusions.

Integration of pQTLs with EHR data from diverse populations enriches discovery of genetic associations in both common disease such as coronary heart disease and more rare disorders such as sarcoidosis and MS. The increasing availability of whole-genome sequencing (WGS) and molecular profiling will continue to aid in the discovery of biomarkers and pathways of complex diseases. Our results highlight the importance of conducting such investigations in diverse populations.

## Methods

### Sex as a biological variable.

All study populations included both males and females, and biological sex was treated as a covariate in all analyses.

The JHS is a community-based longitudinal cohort study of 5,301 self-identified Black or African American individuals from the Jackson, Mississippi, USA, metropolitan statistical area (46). The first exam was conducted from 2000–2004; here we utilized data from that first exam. Second and third exams have also been conducted, with a fourth exam ongoing. We prioritized participants for initial Olink profiling who were also included in the RNA-Seq sample, as previously described (47). Included in the present study are 1,054 individuals selected for proteomics profiling at exam 1, who also have available whole-genome sequencing. MESA recruited 6,814 men and women aged 45 to 84 years at 6 clinical centers across the United States, with the first exam occurring in 2000–2002 and 5 subsequent exams (exam 7 ongoing). Participants self-identified with 1 of 4 race/ethnicity groups: Black, Hispanic, Asian, or White. Included in the present study are 2,120 individuals from exam 1 who have measured proteomics using the Olink platform and have available whole-genome sequencing (48). The JHS participants who underwent Olink proteomic profiling (*n* = 1,040) were selected for the availability of both whole-genome sequencing and blood RNA transcriptomics data. In the MESA cohort, participants were included for proteomic profiling based on the availability of samples for exams 1, 5, and 6 as part of a longitudinal proteomics study. Proteomics data from exam 1 were used for the GWAS. Supplemental Table 1 includes demographics and clinical factors included in this analysis versus the full cohorts, which were overall quite similar.

Whole-genome sequencing (≥30×) for both JHS (discovery) and MESA (replication cohort) is through the National Heart, Lung, and Blood Institute’s (NHLBI’s) Trans-Omics for Precision Medicine (TOPMed) program. We here utilize sequencing data from the freeze10 call set; detailed methods are similar to prior freezes and are available at https://topmed.nhlbi.nih.gov/data-resources/methods

### Proteomic profiling.

The Olink antibody-based platform and technology have been described previously (49). Briefly, pairs of oligonucleotide-labeled antibody probes specifically bind to their respective target proteins. When these probes come close to each other, the oligonucleotides hybridize, and a proximity-dependent DNA polymerization occurs. This generates a unique PCR target sequence. The resulting DNA sequence is then detected and quantified using a microfluidic real-time PCR instrument (Biomark HD, Fluidigm). To ensure data quality and account for variation between runs, internal controls including an extension control and an interplate control are used for normalization. The final output of the assay is presented as Normalized Protein eXpression (NPX) values, which are arbitrary units on a log_2_ scale. Higher NPX values indicate higher protein expression. We removed proteins with a CV of more than 20% from our analyses. Detailed assay validation data, such as detection limits and intra- and interassay precision, can be found on the manufacturer’s website (www.olink.com).

### Statistics.

For genetic analyses, assay values were log-transformed, scaled (mean = 0, SD = 1), batch-corrected in JHS, and adjusted for age, sex, batch, and ancestry-principal components 1 to 10 in each cohort. The resulting residuals underwent inverse rank normalization. We assessed the association between these values and genetic variants using linear mixed-effects models. The models were adjusted for age, biological sex, genetic relationship matrix (as a random effect), and the first 10 principal components. The analysis was performed using the fastGWA model within the GCTA software package (version 1.93.2beta/gcta64) (18). Repeat adjustment was applied to minimize type I error and enhance statistical power (50). Variants with a minor allele count less than 5 within a specific cohort were excluded from the analysis for that cohort. To identify the index or sentinel variants in each association region of each protein, 1Mb regions encompassing each SNP linked to a specific protein were established. Starting with the region housing the variant with the lowest *P* value, overlapping regions were consolidated. This process was iterated until there were no more overlapping regions related to the respective protein. The sentinel variant for each region was determined as the one with the lowest *P* value, and the encompassing region as the pQTL locus. In the JHS cohort, a Bonferroni-adjusted significance threshold of 5 × 10^–8^ was applied for *cis*-variant associations. A stricter Bonferroni correction for *trans*-variation of 7 × 10^–11^ (5 × 10^–8^/670, with 670 being the number of protein principal components explaining 95% variation of protein levels) was utilized for discovery purposes. Sentinel variants were annotated using the FAVOR database (51). Associations with sentinel variants were tested in MESA for validation. A nominal *P* value threshold of less than 0.05 with consistent direction of effect was considered significant for these replication analyses. To perform multiancestry statistical fine mapping of our associations, we conducted fixed effects metaanalyses across JHS and MESA. We fine mapped our significant pQTLs from JHS to identify credible sets of potentially causal variants using SuSiE (52) utilizing individual level LD information from JHS and MESA. We assessed for overlap in our credible sets with credible sets from the recent UK Biobank GWAS (3). In addition, we assessed overlap between eQTL and sQTL credible sets from JHS RNA profiling in individuals with stored PBMCs (53).

For global and local ancestry inference, we estimated the overall proportion of African ancestry (PAA) of each individual, as well as estimated number of African ancestry haplotypes at each genomic location (or local ancestry estimates), using RFMix (54) with 2 reference groups representing European and African ancestry from 1000G. We considered only European and African ancestry reference panels, based on prior work (44) with global/local ancestry inference in JHS and in self-identified African American participants from MESA. Our 1000G reference panel included 503 European samples and 503 African samples. African samples were randomly downsampled to have an equivalent sample size to the European reference panel. We used linear regression to test the association of the level of each protein with the estimated number of African ancestry haplotypes at each genomic location, while controlling for age, sex, and estimated global ancestry. We used the previously described admixture mapping significance threshold for AA participants of 2.1 × 10^–05^ (55) Correcting for 670 principal components explaining 95% variation of protein levels, we got a Bonferroni-adjusted significance threshold of 3.1 × 10^–08^. All association analyses were conducted in PLINK. We defined a “signal region” identified through ancestry mapping as the contiguous region with association *P* values lower than the admixture mapping threshold (3.1 × 10^–08^). We then defined a broader region by extending the signal region to nearby flanking regions 1 M bp or less upstream or downstream from the signal region. We then tested to determine whether previously reported variants within this broader region from a prior single variant WGS analysis in the UK Biobank (3) for the same protein could explain the admixture mapping signal, by adjusting for the previously reported variants to assess whether the signal remained statistically significant. We replicated our JHS findings in the MESA in self-identified Black individuals, focusing on proteins that had a significant admixture mapping signal. We again used linear regression to test the association of local African ancestry at each genomic location with the level of each protein, while controlling for age, sex, and estimated global ancestry. The replication criterion was a nominal significance threshold of 0.05 with the same direction of estimated effect.

### PheWAS.

We examined the phenotypic associations of identified pQTLs through PheWAS in 2 diverse biobanks: BioMe and All of Us. The BioMe biobank is a hospital-based cohort that includes participants recruited from the BioMe Biobank Program (Mount Sinai, New York, USA) from 2007 to the present (15). The current analysis included 53,227 individuals (16,336 individuals of predominantly African ancestry) with EHRs and genetic information. Genotyping of BioMe participants was performed using the global diversity and global screening arrays as previously described. The All of Us Research Program is an ongoing biobank effort collecting data from community-dwelling adults across the United States. We utilized WGS data from the All of Us version 7 dataset (16), from 165,567 individuals with WGS and EHR data available. For both BioMe and All of Us, ICD-9 and 10 codes were grouped into phecodes (56) and treated as dichotomous outcomes. Phecodes with fewer than 100 cases were excluded from subsequent analyses, resulting in 845 phecodes present in both BioME and All of Us. Firth’s logistic regression adjusted for age, sex, and 16 principal components of ancestry was used to examine the association of each individual pQTL with each phecode. When a phecode was included through both cohorts, SNP-phecode associations were metaanalyzed using METAL (57). Significance was determined at a FDR of less than 5% from metaanalysis, concordant direction of effects across BioME and All of Us, and nominal significance (*P* < 0.05) in both cohorts. BioME also contains data on hospital-based laboratory measurements, reflecting intermediate phenotypes, for all individuals. All laboratory measurements were inverse rank normalized. We performed linear regression, adjusted as above, to test the association between each pQTL and 1,686 continuous laboratory tests. A FDR of less than 5% was used to determine significance. All PheWAS were performed in both the full cohorts and self-identified Black or African American individuals only as a sensitivity analysis. An interactive web tool to browse summary statistics from the GWAS and PheWAS analyses is made available at https://bidmc-cardiology-2024.shinyapps.io/pqtl_phewas_explorer/ To examine the potential novelty of identified pQTL-phenotype relationships, we queried PheWAS results against the GWAS catalog (58) (download date September 2023). We collated significant GWAS results from a 250 Kb range around each variant and manually checked for SNP-phenotype matches to significant PheWAS associations. Associations were annotated as novel if there were no variants +/– 250 kb of the pQTL in the GWAS catalog associated with the same phenotype found in PheWAS.

### Study approval.

The JHS study was approved by the Jackson State University, Tougaloo College, and University of Mississippi Medical Center Institutional Review Boards, and all participants provided written, informed consent. All MESA participants provided written, informed consent, and the study was approved by the Institutional Review Boards at The Lundquist Institute (formerly Los Angeles BioMedical Research Institute) at Harbor-University of California, Los Angeles, Medical Center, University of Washington, Wake Forest School of Medicine, Northwestern University, University of Minnesota, Columbia University, Johns Hopkins University, and University of California, Los Angeles.

### Data availability.

Data utilized here are available either in dbGaP (JHS: phs000964/phs002256; MESA: phs001416/phs000209), or for newly generated proteomics data that are being submitted to dbGaP, through study coordinating centers (JHS: https://www.jacksonheartstudy.org/ and MESA: https://www.mesa-nhlbi.org/). Summary results are made publicly available at: https://bidmc-cardiology-2024.shinyapps.io/pqtl_phewas_explorer/ Values for all data points in graphs are reported in the Supporting Data Values file. Analytic code is available upon request.

## Author contributions

All authors read and approved the manuscript. UAT and JLB share first authorship in the given order based on their relative contributions to the project. UAT and JLB conceived and designed the study, performed data analyses in MESA, JHS, and All of Us, and wrote the manuscript. DEC, EK, SD, BT, YI, and MG performed data analysis and reviewed the manuscript. MDB, JMR, ZZC, PR, DHK, TS, MEH, TJW, and AR reviewed and provided valuable intellectual input on the manuscript. LF oversaw and performed proteomic profiling in MESA and JHS. LE, PD, WCJ, and RPT oversaw sample and dataset distribution and revised the manuscript. KDT, YL, XG, YDIC, AWM, DJ, and PN reviewed analyses and provided important intellectual input on the manuscript. JIR and REG secured funding for proteomic profiling in MESA and JHS. JIR, SSR, JGW, LMR, and REG supervised all analyses and revised the manuscript.

## Supplementary Material

Supplemental data

ICMJE disclosure forms

Supplemental tables 1-12

Supporting data values

## Figures and Tables

**Table 1:**
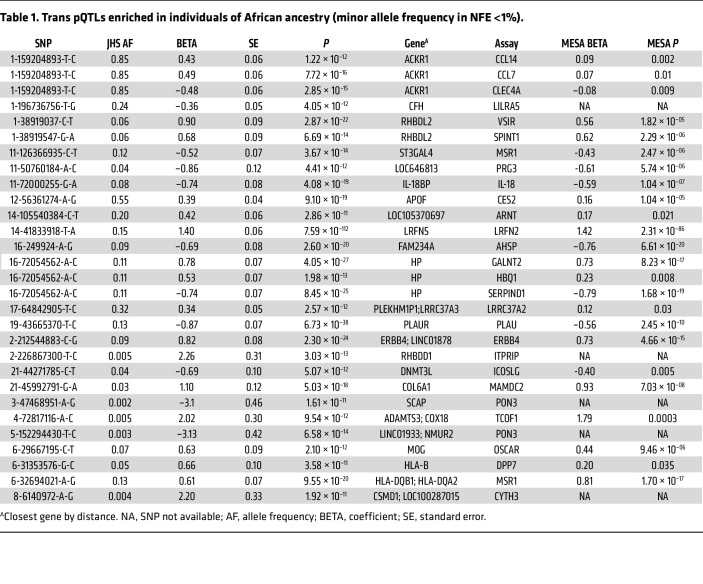
Trans pQTLs enriched in individuals of African ancestry (minor allele frequency in NFE <1%).

**Table 2:**
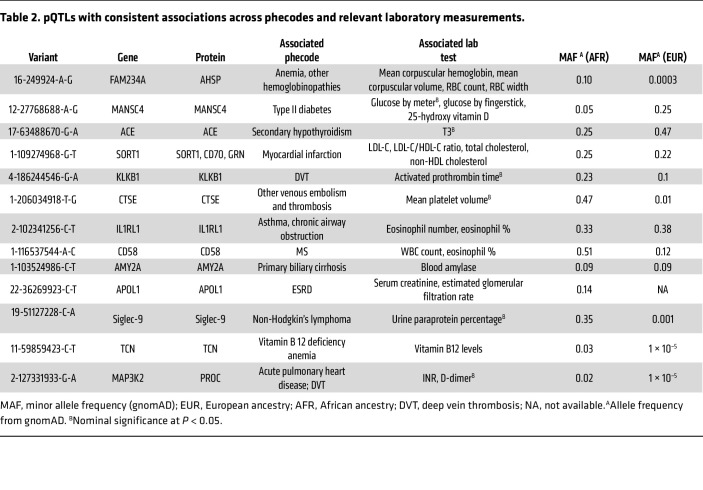
pQTLs with consistent associations across phecodes and relevant laboratory measurements.

**Table 3:**
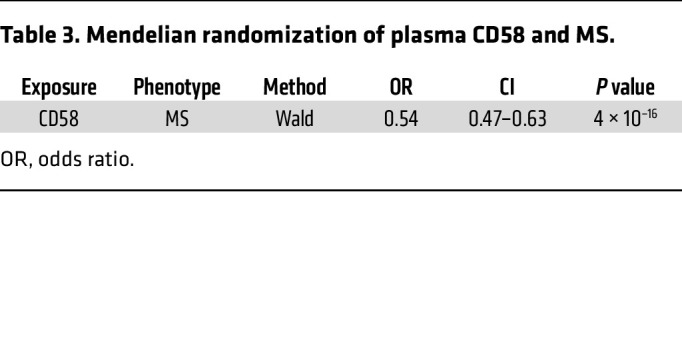
Mendelian randomization of plasma CD58 and MS.

## Version 1 09/24/2024

In-Press Preview

## Version 2 11/01/2024

Electronic publication
